# Egas Moniz: A Centennial Tribute to Innovation

**DOI:** 10.1161/SVIN.124.001414

**Published:** 2024-11-05

**Authors:** Fawaz Al‐Mufti

**Affiliations:** ^1^ Westchester Medical Center at New York Medical College Valhalla NY

About the Technique: Op Art (using geometric shapes to create illusion and movement)

About the Art: In commemoration of the American Heart Association's centennial anniversary, I reflect on a century of advancements in cardiovascular health and interventional neurosciences. This milestone also marks nearly a hundred years since the discovery of cerebral angiography, a pivotal moment in medical history.

Central Image: This artwork pays homage to Egas Moniz, the father of neuroangiography, whose groundbreaking work, begun almost a century ago, revolutionized our understanding of cerebral vasculature and paved the way for precise diagnostic imaging techniques in neurology. Despite its intricate nature, the cerebral vasculature possesses a remarkable beauty that captivates both the eye and the imagination.

Using undiluted India ink on Bristol board, the artwork aptly exemplifies the complexity and intricacies of the cerebral vasculature. With its rapid drying nature, requiring swift and precise strokes, akin to the finesse demanded in interventional neurosciences, almost 5000 tiny triangular and rhomboidal black shapes are meticulously crafted. Each element intricately weaves together to replicate the shadows and contours of the brain's intricate network of blood vessels. Through this meticulous craftsmanship, we pay homage to the legacy of Egas Moniz and his profound contributions to the field of neuroangiography.

Moniz's journey was one of professional achievement, personal struggle, and resilience. Born António Caetano de Abreu Freire de Resende in Avanca, Portugal, he pursued a career in medicine, eventually becoming a renowned neurology professor at the University of Lisbon. His unwavering commitment to medical innovation led to the development of the first cerebral angiogram, a groundbreaking achievement that earned him international recognition and Nobel Prize nominations.

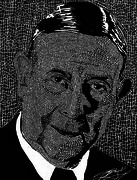

Beyond his medical contributions, Moniz embodies the multifaceted nature of interventional neuroscientists, driven by diverse passions and pursuits. From his early involvement in politics to his prolific writings on diverse subjects ranging from Portuguese literature to sexology, Moniz's intellectual curiosity knew no bounds. Despite facing adversity, including a life‐altering gunshot wound from a patient with schizophrenia, Moniz continued to make enduring contributions to medicine until his passing in 1955.

As we honor the legacy of Egas Moniz and celebrate the American Heart Association's and angiography's centennial anniversary, may this artwork serve as a poignant reminder of the relentless pursuit of knowledge and innovation in the field of vascular and interventional neurosciences.

